# The Relationship Between Age and the Morphology of the Crystalline Lens, Ciliary Muscle, Trabecular Meshwork, and Schlemm’s Canal: An *in vivo* Swept-Source Optical Coherence Tomography Study

**DOI:** 10.3389/fphys.2021.763736

**Published:** 2021-11-19

**Authors:** Zhangliang Li, Ziqi Meng, Wenyong Qu, Xiuyuan Li, Pingjun Chang, Dandan Wang, Yune Zhao

**Affiliations:** ^1^Eye Hospital and School of Ophthalmology and Optometry, Wenzhou Medical University, Wenzhou, China; ^2^National Clinical Research Center for Ocular Diseases, Wenzhou, China; ^3^Eye Hospital of Wenzhou Medical University Hangzhou Branch, Hangzhou, China

**Keywords:** anatomy, age, Schlemm’s canal, ciliary muscle, crystalline lens

## Abstract

**Purpose:** To evaluate the effects of age on the morphologies of the crystalline lens, ciliary muscle (CM), Schlemm’s canal (SC), and trabecular meshwork (TM) using swept-source optical coherence tomography (SS-OCT).

**Methods:** Images of the crystalline lens and iridocorneal angle were obtained in healthy participants’ eyes using SS-OCT. Morphological parameters of the crystalline lens, CM, and TM/SC were measured, and the relationship between these parameters and age was evaluated.

**Results:** A total of 62 healthy participants were enrolled, with an age range of 7–79 years. With adjustments for the effects of axial length and sex, both the nasal and temporal SC cross-sectional areas (CSA) and the cross-sectional area of the CM (CMA), distance from the scleral spur to the inner apex of the ciliary muscle (IA-SS), and nasal SC volume were negatively correlated with age (*P* ≤ 0.041). Meanwhile, the lens thickness (LT) (*P* < 0.001) and lens vault (LV) (*P* < 0.001) were positively correlated with age, and the radius of the curvature of the anterior lens (ALR) was negatively correlated with age (*P* < 0.001).

**Conclusion:** Increasing age was associated with a thicker crystalline lens, a steeper anterior lens curvature, an anteriorly located and smaller CM, and a narrower SC.

**Clinical Trial Registration:** https://register.clinicaltrials.gov/prs/app/action/Select Protocol?sid=S000A3JZ&selectaction=Edit&uid=U00019K7&ts=4&cx=-c5xxp8, identifier [NCT04576884].

## Introduction

Glaucoma is the most common irreversible cause of blindness. The prevalence of primary open-angle glaucoma (POAG) has been shown to increase with age (Kreft et al., 2019). Due to aging of the general population, the number of people with glaucoma is projected to increase to 111.8 million worldwide by 2040 (Tham et al., 2014).

The elevated intraocular pressure (IOP) of glaucoma is due in part to pathological changes in the pathway for aqueous humor outflow, with 75–80% of the aqueous humor flowing out through the conventional trabecular meshwork (TM) and Schlemm’s canal (SC) pathway. Changes in this pathway play a major role in determining IOP (Tamm, 2009). It has been proven that the SC is significantly smaller in patients with glaucoma than in normal subjects (Yan et al., 2016; Tandon et al., 2017). The decreasing SC size in the elders (Chen et al., 2018; Zhao et al., 2018) might result from several factors, such as an age-related reduction in the number of SC cell count (Ainsworth and Lee, 1990), and a decreases in aqueous humor production with age (Becker, 1958). Though previous studies have proposed the effect of age on the morphology of crystalline lens, ciliary muscle (CM) or TM/SC solely (Richdale et al., 2013, 2016; Chen et al., 2018; Zhao et al., 2018; Fernández-Vigo et al., 2020; Xie et al., 2020), the effect of age on these structures as an entity has not been fully studied. Evaluation of age-related changes in the crystalline lens, CM and TM/SC of human eyes might be helpful in understanding why the incidence of POAG increases with age.

Optical coherence tomography (OCT) is a non-invasive imaging system for biological tissue scanning. The CASIA OCT (Tomey, Nagoya, Japan) is a commercially available swept-source optical coherence tomography (SS-OCT) designed specifically for anterior-segment imaging. In CASIA SS-1000 OCT images, the scleral spur is visualized best, followed by Schwalbe’s line. However, images of the trabecular aqueous outflow pathway are often suboptimal, with an unclear demarcation of SC lumens (McKee et al., 2013). The CASIA2 is the newest commercially available SS-OCT system. Compared with the CASIA SS-1000, images of anterior chamber angle structures have a higher resolution with the CASIA2 because B-scan images consist of four superimposed images.

The purpose of this study was to observe the morphologies of the lens, CM, and TM/SC using the CASIA2 SS-OCT system and to investigate the relationship between the morphologies of these structures and age.

## Materials and Methods

### Participants

Our study was approved by the Ethics Committee of the Eye Hospital of Wenzhou Medical University and followed the tenets of the Declaration of Helsinki. All participants were recruited from the outpatient clinic of the Eye Hospital of Wenzhou Medical University from July 2020 to September 2020, and all of them and/or their legal guardians signed informed consent forms with detailed explanations about the nature of this study.

We exclusively enrolled normal participants without any history of ocular surgeries or diseases (except for cataracts and refractive errors), particularly those that could affect the structures of the aqueous outflow pathway, such as lens dislocation, uveitis, intraocular neovascular diseases, glaucoma, a shallow anterior chamber, or anterior segment congenital anomalies. Ocular conditions that could affect the image quality, such as pterygium or corneal diseases, were also excluded.

Slit-lamp biomicroscopic examinations and fundus examinations were performed by a practical ophthalmologist (LZL). Participants underwent comprehensive ophthalmologic examinations, including assessments of IOP (non-contact tonometer; TX-F; Cannon, Tokyo, Japan), objective refraction (autorefractometer; KR-8900; Topcon, Tokyo, Japan), and axial length (AL) (IOL Master 700, Carl Zeiss Meditec AG). All examinations were performed by the same operator. Data from the right eye were collected for analyses.

### Optical Coherence Tomography Data Acquisition and Processing

All participants underwent SS-OCT (CASIA 2; Tomey, Nagoya, Japan) examinations specifically designed for anterior-segment imaging using a 1,310-nm wavelength, a scan speed of 50,000 A-scans per second, and an axial resolution of less than 10 μm. Before using the CASIA2 for the first time, the optical calibration was performed using model eyes.

All SS-OCT tests were performed under the same illumination of indoor light conditions because the pupillary reaction can alter the structure of the angle. With consideration of the influence of accommodation on parameters of the lens, the refractive error was input and corrected by the built-in program.

During the test, the participants were asked to stare at the built-in light ahead. Cross-sectional images were acquired using a biometry-type lens protocol, and the radius of the curvature of the anterior lens (ALR), the lens thickness (LT), and the lens vault (LV) were obtained using the built-in software and measurement tools provided by the manufacturer. One operator scanned three sets of images, and average values were used for analyses.

Thereafter, participants were asked to stare at left or right built-in fixation lights to ensure that the iridocorneal angle was centered in the instrument’s field of view. The morphology of CM and TM/SC varies in different quadrants of the eye (Kagemann et al., 2010; Sheppard and Davies, 2010; Fernandez-Vigo et al., 2017; Li et al., 2017; Chen et al., 2018; Zhao et al., 2018; Fernández-Vigo et al., 2020). In this study, the scans were performed independently for the temporal and nasal quadrants (at the 3 and 9 o’clock positions) using the angle HD type. The iridocorneal angle was scanned in a cubic area with a length, width and height of 12, 4, and 14 mm, respectively. Sixteen B-scans were horizontally arranged on the length of the cuboid, and each B-scan had 800 A-scans. It took 1.15 s to capture all images. Every B-scan image consisted of four images automatically superimposed on each other to decrease the speckle noise. To improve the quality of the image, three sets per nasal and temporal areas were taken, and the set with the best quality was selected for further processing.

**FIGURE 1:**
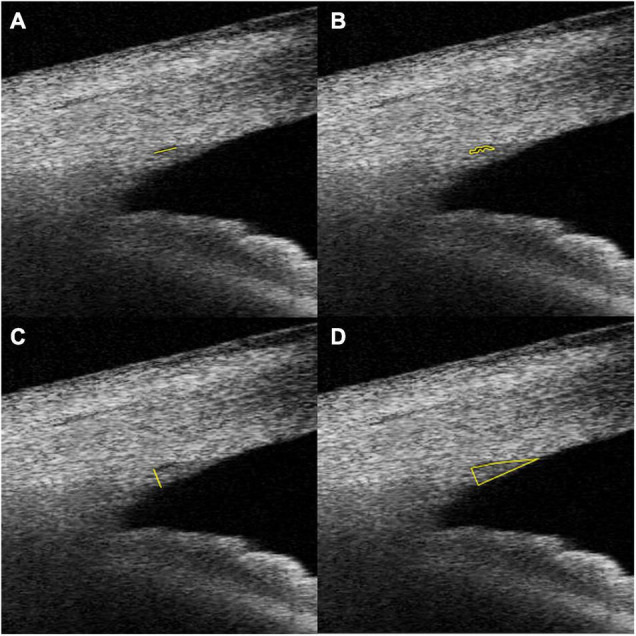
Examples of SC diameter **(A)**, CSA **(B)**, TM height **(C)**, and TM area **(D)** measurements. SC, Schlemm’s canal; CSA, Schlemm’s canal cross-sectional area; TM, trabecular meshwork; TMA, cross-sectional area of trabecular meshwork.

**FIGURE 2:**
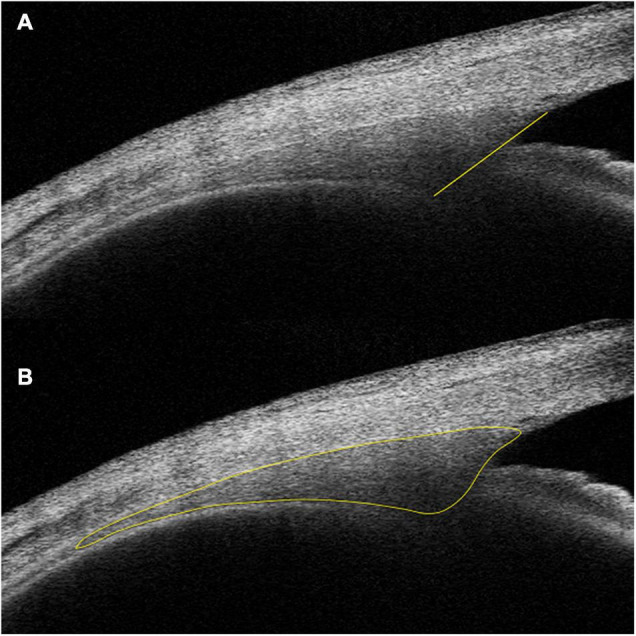
Examples of IA-SS **(A)** and CMA **(B)** measurements. IA-SS, scleral spur to the inner apex of the ciliary muscle; CMA, cross-sectional area of the ciliary muscle.

Schlemm’s canal was defined as a thin, black, lucent space on the image. We selected eyes with an observable SC in at least 50% of the 16 B-scans in both sides. For images with an observable SC, the SC diameter and area, TM height and area, CM area (CMA), and the distance between scleral spur and inner apex of ciliary muscle (SS-IA) were quantified manually using ImageJ software (http://imagej.nih.gov/ij/; provided in the public domain by the National Institutes of Health, Bethesda, MD, United States), and average values were calculated. Every image was magnified by 200% for measuring the SC diameter and area. The SC diameter was measured from the posterior to the anterior end points of SC (Figure 1A). The SC cross-sectional area (CSA) was drawn as a long circular region inside of the SC outline (Figure 1B). The volume of SC was the cumulative total of the products of CSA and the scanning gap between each B-scan (250 μm). The scleral spur appeared as a hyperreflective point between the TM and the ciliary body, and Schwalbe’s line was defined as the border between the hyperreflective corneal endothelium and the hyporeflective TM. When measuring TM parameters, the image was magnified by 150%, and the TM height was defined as the distance between the posterior endpoint of SC and the scleral spur (Figure 1C). We defined the TM area (TMA) as the area enclosed by lines connecting the scleral spur, posterior endpoint of SC, and Schwalbe’s line (Figure 1D). The image was then magnified by 50% to measure CM parameters. The CM was identified as a hyporeflective triangle-like structure, with the inner surface corresponding to the interface between the vitreous cavity and the ciliary body pigmented epithelium (hyperreflective), the outer surface between the sclera (hyperreflective) and the CM (hyporeflective), and the anterior chamber boundary as the base layer. The posterior limit was the point where hyperreflective inner and external CM surfaces have minimal separation posteriorly and become parallel thereafter. The drawing outline of the hyporeflective triangle-like structure was recorded as the CMA (Figure 2B). The SS-IA was defined as the distance from the scleral spur to the inner apex of the CM (Figure 2A).

All parameters were measured by a single blinded ophthalmologist (MZQ) who was not informed of age and ocular characteristics of participants. To assess the repeatability regarding the image analysis using ImageJ, measurements were repeated within two separate time in twelve randomly chosen eyes, and the coefficient of variation (CV) and the intraclass correlation coefficient (ICC) were calculated.

### Statistical Analysis

Statistical analysis was performed using SPSS (version 21.0; SPSS Inc., Chicago, Illinois, United States). The normality of the data was assessed using the Kolmogorov-Smirnov test. The paired two-sample *t*-test was used to analyze the differences in parameters between the nasal and temporal quadrants. Pearson analysis and simple linear regression were used to analyzed the correlation between parameters and age. Multivariable linear regression analysis was performed to evaluate the associations between parameters of the lens, CM, TM/SC and age, with adjustments for the effects of AL and sex. The CV was defined as the ratio of the SD to the mean of a measurement. The ICC was calculated by using a two-way mixed effect model. All tests were two-tailed, and statistical significance was defined as a *P*-value of <0.05.

## Results

The study initially included 78 participants. Sixteen participants were then excluded because the borders of their SC lumens were not clearly visible on more than 50% of one set (16 B-scans). Therefore, 62 participants (62 eyes) aged 7–79 years were selected for the final analysis. Table 1 shows the demographic and ocular characteristics of the participants. Averagely, 12.55 ± 2.27 B-scans were measured in each selected set.

**TABLE 1:** Demographic and ocular characteristics of study participants.

Parameter	Participants
n	62
Gender, male/female	23/39
	**Mean ± SD, range**
Age, y	40.3 ± 23.9, 7–79
SE, D	−1.48 ± 3.19, −9.50 to 7.50
IOP, mm Hg	13.1 ± 2.8, 7.9–18.7
AL, mm	23.86 ± 1.20, 21.34–26.04

*SE, spherical equivalent; IOP, intraocular pressure; AL, axial length.*

The measurement of CSA, SC diameter, TM area, TM height, CMA and SS-IA showed good repeatability with the ICC ≥ 0.8 (all *P* < 0.01). The ICC value was 0.667 for the SC volume. The CVs of the SC diameter, CSA, SC volume, TM area and TM height were 20.0, 26.5, 35.6, 16.7, and 13.0%, respectively. Acceptable repeatability and reproducibility of the lens morphologies using this SS-OCT has been reported in our previous study (Li et al., 2021).

### Lateral Symmetry Analysis

Compared with parameters in the temporal quadrant, the SC volume (*P* = 0.036) and CMA (*P* < 0.001) were significantly smaller in the nasal quadrant, while the TMA (*P* < 0.001) and TM height (*P* = 0.001) were significantly larger (Table 2). We found no significant differences of other parameters between two quadrants.

**TABLE 2:** Comparison of parameters between nasal and temporal quadrants.

Parameter	Nasal	Temporal	*P*-value
SC diameter, μm	187.3 ± 32.7	190.1 ± 41.5	0.519
CSA, μm^2^	3101.8 ± 685.6	3240.6 ± 871.3	0.152
SC volume, μm^3^	9.57 × 10^6^ ± 2.89 × 10^6^	10.48 × 10^6^ ± 3.81 × 10^6^	0.036
TMA, μm^2^	60282.0 ± 9655.0	55600.0 ± 8637.8	<0.001
TM height, μm	213.1 ± 28.5	207.7 ± 23.8	0.001
CMA, μm^2^	1.07 × 10^6^ ± 0.20 × 10^6^	1.13 × 10^6^ ± 0.23 × 10^6^	<0.001
IA-SS, μm	984.9 ± 153.4	982.1 ± 177.4	0.811

*SC, Schlemm’s canal; CSA, Schlemm’s canal cross-sectional area; TM, trabecular meshwork; TMA, cross-sectional area of the trabecular meshwork; CMA, cross-sectional area of the ciliary muscle; IA-SS, scleral spur to the inner apex of the ciliary muscle.*

### Relationship Between Parameters and Age

**FIGURE 3:**
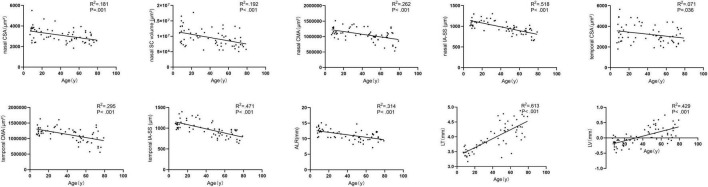
Correlations between nasal, temporal, and lenticular parameters and age.

Figure 3 showed the correlation between parameters and age. We noted significant correlation between CSA, CMA, IS-AA, lenticular parameters (ALR, LT, and LV) and age (All *P* < 0.05). In multivariable linear regression model, the nasal CSA (*P* = 0.002), SC volume (*P* = 0.002), CMA (*P* < 0.001), and IA-SS (*P* < 0.001) and the temporal CSA (*P* = 0.041), CMA (*P* < 0.001), and IA-SS (*P* < 0.001) were significantly negatively correlated with age after adjustments for AL and sex. Meanwhile, the ALR (*P* < 0.001) was negatively correlated with age, and the LT (*P* < 0.001) and LV (*P* < 0.001) were positively correlated with age. Other parameters showed non-significant correlations with age (Table 3).

**TABLE 3:** Correlations between nasal, temporal, and lenticular parameters and age with adjustments for axial length and sex.

Parameter	*P*-value	Unstandardized coefficient β	95% CI for coefficient β
**Nasal**			
CSA, μm^2^	0.002	–11.222	−18.198, −4.247
SC volume, μm^3^	0.002	–47507.076	−76517.365, −18496.787
CMA, μm^2^	< 0.001	–3713.791	−5403.769, −2023.812
IA-SS, μm	<0.001	–4.318	−5.311, −3.326
**Temporal**			
CSA, μm^2^	0.041	–9.941	−19.483, −0.398
SC volume, μm^3^	0.086	–36759.550	−78880.537, 5361.437
CMA, μm^2^	<0.001	–4356.348	−6067.868, −2644.829
IA-SS, μm	<0.001	–4.696	−5.951, −3.442
**Lenticular**			
ALR, mm	<0.001	–0.038	−0.054, −0.022
LT, mm	<0.001	0.015	0.012, 0.018
LV, mm	<0.001	0.007	0.005, 0.009

*CI, confidence interval; SC, Schlemm’s canal; CSA, Schlemm’s cross-sectional area; CMA, cross-sectional area of ciliary muscle; IA-SS, scleral spur to the inner apex of the ciliary muscle; ARL, radius of the curvature of the anterior lens; LT, lens thickness; LV, lens vault.*

## Discussion

In this study, the trabecular meshwork and Schlemm’s canal was well imaged by the swept-source optical coherence tomography system (CASIA 2) the in the majority of participants (62 of 78 participants) and the morphological parameters manually measured by the ImageJ software showed good repeatability. In the lateral symmetry analysis, we discovered the SC volume, CMA, TMA, and TM height were significantly different between the nasal and temporal quadrants. In the multivariable linear regression model, the lenticular parameters (ALR, LT, and LV), nasal CSA, SC volume, IA-SS, CMA and temporal CSA, IA-SS, and CMA were significantly associated with age, with adjustment of AL and sex.

In previous studies, there was no consensus on the appropriate assessment methodology for the CSA. Many researchers (Gold et al., 2013; Hong et al., 2013; Zhao et al., 2018) used a single-line scan protocol to acquire limbus images until a clear view of the SC is obtained. In this way, the largest viewable region of SC is most likely measured. Other researchers (Zhao et al., 2016; Chen et al., 2018) have chosen a volumetric scan protocol, but have only selected three B-scans from each site for measurements. Since two adjacent B-scans can have different cross-sectional areas, averaging the CSAs measured in all B-scans may be the gold standard to evaluate the sectional morphology of SC (Kagemann et al., 2014). In our study, the SC, TM, and CM parameters were quantified manually in images with an observable SC, and we calculated the volume of the SC. By this method, we avoid variability in the morphology resulting from inconsistent scanning positions and provided more reliable results when analyzing the parameters. In addition, the CASIA 2 could visualize the anterior segment from the anterior surface of cornea to the posterior surface of crystalline lens. It facilitated evaluating of the TM/SC, CM, and the entire lens from the same group of participants using the same instrument.

The symmetry in morphology between the nasal and temporal parameters remains controversial. Our results demonstrated non-significant differences in the SC diameter and the CSA between the nasal and temporal quadrants, which is consistent with previous studies (Shi et al., 2012; Chen et al., 2018; Fernandez-Vigo et al., 2019). In contrast, other authors have reported significant differences in the CSA between the nasal and temporal sections, with a larger CSA in either the temporal (Zhao et al., 2018) or nasal (Kagemann et al., 2010; Li et al., 2017) sections. Calculating the SC volume may magnify the difference in the CSA, and we found that the SC volume was significantly larger in the temporal quadrant. In our study, the TMA (*P* < 0.001) and TM height (*P* = 0.001) were significantly larger in the nasal quadrant. Other investigators, however, have not reported a difference between nasal and temporal TM values (Fernandez-Vigo et al., 2017; Chen et al., 2018). In most studies, a significantly larger CMA and thicker CM in the temporal quadrant was detected (Sheppard and Davies, 2010; Fernández-Vigo et al., 2020). In current study, the CMA (*P* < 0.001) was also significantly larger in the temporal quadrant. Similarly to Sheppard et al.’s study, we found no significant difference between nasal and temporal IA-SS measurements (Sheppard and Davies, 2011). This finding is not consistent with the result of a study by Xie et al. (2020) in which the mean IA-SS on the nasal side was significantly greater than on the temporal side. This discrepancy may partly be ascribed to the different imaging devices and measurement methods used. Xie et al. (2020) measured the IA-SS in the images along the horizontal meridian across the corneal vertex, while we averaged the values of IA-SS measured in all the 16 B-scans in a cubic scanning area.

In a histopathologic study performed on donor eyes, the authors found a significant reduction in the size of SC with age, which is consistent with our results (Ainsworth and Lee, 1990). Similarly, Chen et al. (2018) noted a clear decline in the SC diameter and CSA as age increased, with a linear correlation analysis indicating a significant age-dependent tendency (*P* < 0.001) using the CASIA SS-1000 OCT. Zhao et al. (Zhao et al., 2018) divided participants into four groups (16–20 years, 21–40 years, 41–60 years, and 61–80 years) and found that the nasal CSA was significantly smaller in the 61–80-years group. Studies have also reported that the crystalline lens thickness increases, the anterior lens curvature steepens, and the IA-SS and CMA decrease with age (Richdale et al., 2013, 2016; Fernández-Vigo et al., 2020; Xie et al., 2020). Previous studies have reported that the SC size increases in response to accommodation efforts and shrinks with cycloplegia in children (Daniel et al., 2018; Xiang et al., 2020). In older adults, the tonic accommodation and amplitude of accommodation declined (Mordi and Ciuffreda, 1998; Strenk et al., 2005; Shao et al., 2015). As known, the elastin fibers of the CM tendons are connected to the elastin network within the trabecular lamellae, and the CM tendon fiber density is most dense near the juxtacanalicular tissue (JCT) (Park et al., 2016). Based on this anatomic connection between the accommodation involved organs (crystalline lens and ciliary body) and aqueous humor outflow pathway (trabecular meshwork and Schlemm’s canal), we designed the current study to investigate how this anatomic entity changes with age. We found the steepening anterior crystalline lens surface concurrent with shrinking and anteriorly located CM and shrinkage of SC as age growing, and this might partly explain the phenomenon of increasing prevalence of POAG with age (Kaufman et al., 2019). Since all the measurements were performed when the eye is relaxed in our study, whether losing accommodation plays a role in the role of developing glaucoma needs to be further studied.

Our study had certain limitations. First, we only measured the SC and TM parameters in the nasal and temporal quadrants. Variations in SC and TM parameters in the superior and inferior quadrants were not studied. Second, some undistinguishable lumens of SC were considered to be absent and not included in the final analysis. Therefore, the calculated volumes may not be identical to the true values. Third, the IOP in our study was obtained using a non-contact tonometer rather than a Goldmann applanation tonometer, and the measuring time varies in a day. These factors might affect the accuracy of the IOP measurement, as well as our analysis of the relationship between the IOP and TM/SC parameters. Whether changes in TM/SC parameters can influence the IOP requires further research.

## Conclusion

In conclusion, increasing age was associated with a thicker crystalline lens, a steeper anterior lens curvature, an anteriorly located and smaller CM, and a narrower SC. Further studies are needed to investigate whether the changes of lens and CM directly correlate with TM/SC changes and whether these aqueous outflow changes over time can increase the risk of glaucoma.

## Data Availability Statement

The raw data supporting the conclusions of this article will be made available by the authors, without undue reservation.

## Ethics Statement

The studies involving human participants were reviewed and approved by the review board of the Eye Hospital of Wenzhou Medical University. The patients/participants provided their written informed consent to participate in this study.

## Author Contributions

ZL designed the study and was a major contributor to writing the manuscript. ZM was a major contributor to writing the manuscript and analyzed and interpreted the data. WQ and XL collected the data. PC and DW critically revised the manuscript. YZ made substantial contributions to the design of the work. All authors contributed to the article and approved the submitted version.

## Conflict of Interest

The authors declare that the research was conducted in the absence of any commercial or financial relationships that could be construed as a potential conflict of interest.

## Publisher’s Note

All claims expressed in this article are solely those of the authors and do not necessarily represent those of their affiliated organizations, or those of the publisher, the editors and the reviewers. Any product that may be evaluated in this article, or claim that may be made by its manufacturer, is not guaranteed or endorsed by the publisher.
